# Weight perceptions of parents with children at risk for diabetes

**DOI:** 10.1186/1756-0500-5-47

**Published:** 2012-01-20

**Authors:** Eva M Vivian, Tara L Becker, Aaron L Carrel

**Affiliations:** 1 School of Pharmacy, University of Wisconsin-Madison, 1036 Rennebohm Hall, Madison, WI 53705, USA; 2 University of California Los Angeles Graduate Division, 1237 Murphy Hall, Los Angeles, CA 90095-1419, USA; 3 Department Pediatrics, Clinical Science Center, School of Medicine and Public Health, 600 Highland Ave, Box 4108, Madison, WI 53792, USA

## Abstract

**Background:**

The growing epidemic of obesity and diabetes among African American, Latino American, and Native American children in the United States has led to increasing focus on strategies for prevention. However, little is known about the perceptions toward weight, nutrition, and physical activity among these youth. This pilot study explored the perceptions of body weight among overweight and obese children and their parents.

**Results:**

Thirty eight children, ages 8-16 years who were enrolled in a diabetes prevention study were surveyed to assess their perception of their weight. Nearly all (84%) of the children were obese. When asked whether they considered themselves to be overweight, African-American children were less likely to report that they were overweight than other children (33% vs. 80% of other children, *p *= 0.01). The parents of these children (n = 29) were also surveyed to assess their perception of their child's weight. The parents of two-thirds (65%) of the children reported that the child was overweight, while the rest reported their child was underweight or the right weight. African-American parents were less likely to report that their child's weight was unhealthy compared to other parents (46% vs. 77%, *p *= 0.069).

**Conclusions:**

This study's findings indicate that future intervention efforts should assess children's and parents' awareness of obesity and diabetes risk and these factors should be considered when developing prevention interventions for families with youth at risk for diabetes in underserved communities.

## Background

It is now estimated that nearly one out of every six overweight youth has pre-diabetes [1]. Overweight adolescents with type 2 diabetes mellitus (T2DM) are at risk of developing heart disease and other diabetes related complications before the age of 35. The burden of diabetes falls disproportionally on ethnic minority youth, particularly Native Americans, Hispanic/Latino Americans, and African Americans [2-4]. For example, nearly 50% of African American children born in the United States in 2000 are expected to develop diabetes in their lifetime [5]. These alarming figures, combined with the increasing representation of ethnic minorities in our country will result in enormous personal, societal, and economic costs for many decades. Strategies to address this problem are needed immediately, as prevention of diabetes is far preferable to treatment.

Recently research related to childhood obesity and chronic disease prevention focuses on interventions to improve the child's nutritional and physical activity patterns [6,7]. Although this research is beneficial, there is a body of literature suggesting that parents often underestimate their child's weight [8-14] thus indicating a need to explore environmental and social issues related to weight perception.

Parental perceptions of a child's weight may influence how a parent feeds their child [4,8]. One study reported that parents who accurately perceived their child's weight were more likely to believe their child was at risk for type 2 diabetes [13]. Programs targeting children at risk may be unsuccessful if parents do not recognize that the condition exists or the risk it poses to the child's health [8]. Unless parents recognize that a child has an elevated weight, the support needed for a child to achieve a healthy body weight may be lacking [9-14]. Prevention programs which increase parental awareness of their child's weight status may help parents correctly perceive their child's weight status and may lead to behavioral changes. The purpose of this study is to examine the parental perception of their child's body and the child's perception of his or her body weight.

## Methods

We conducted a self-administered survey of 29 parents and 38 children who were enrolled in a diabetes prevention program during month 3 of the 12 month program. The diabetes prevention program was conducted in an ethnically diverse community that consist of 35% Latino Americans, 33% African Americans 10% Asians, 2% American Indians and/or Alaskan Natives, and 20% whites. We selected this community in order to identify families with youth who were at highest risk of developing type 2 diabetes [15].

All the children had at least 2 or 3 risk factors for type 2 diabetes (T2DM) [overweight (body mass index ≥ 85 percentile for age and sex), family history of type 2 diabetes in 1st or 2nd degree relative, ethnicity, or signs of insulin resistance (acanthosis nigricans, hypertension, polycystic ovarian syndrome, or conditions associated with insulin resistance)] at the time of enrollment in the study [16].

A nurse certified diabetes educator (CDE) or pharmacist CDE obtained blood pressure, waist circumference and weight of each child at the time of enrollment in the diabetes prevention study. The child's weight and height was measured with the child in a standing position, wearing light clothing, and without shoes. The child's weight was measured using a digital electronic scale (Conair Body Analysis Weight Tracker Scale Model CON WW89T), and height was measured using a portable stadiometer. BMI was calculated as weight (in kilograms) divided by height (in meters) squared.

The children were asked their perception of their weight. Children were asked if they felt they were overweight (yes or no). Children were also asked their level of agreement (disagree, neutral, agree) with statements "I worry that my weight is unhealthy" and "I worry about becoming overweight".

The parents were asked to report their perception of their child's weight (underweight, about right weight, or overweight). Outcome variables were adapted from the Risk Perception Survey for Developing Diabetes [17]. Parents rated their level of agreement (disagree, neutral, agree) with statements "I worry that my child will become overweight" and "I worry that my child's weight is unhealthy".

Parental consent and child assent was obtained from parents and children prior at the time of enrollment in the study. The study was approved by the Committee for Protection of Human Subjects at the University of Wisconsin-Madison.

### Statistical analysis

All intermediate computations, statistical analyses, and graphical presentations were produced using Stata v11.2 (StataCorp, College Station, TX). Bivariate comparisons were analyzed using Fisher's exact test. Agreement between parent and child assessments of the child's health behaviors was assessed using Cohen's kappa.

## Results

### Child's attitudes about weight

Thirty eight children between the ages of 8 and 16 years, with a mean age of 12 years (± 2.2) and predominantly female (≥ 60%) completed the survey. Twenty two children (57.9%) self-reported as Hispanic/Latino American, 13 as African American (34.2%) and 3 as Native American (7.9%). Nearly all (84%) of the children were obese, 5.3% overweight, and 10.5% normal weight. There were no differences in BMI group by race.

African-American children were less likely to report that they were overweight than other children (33% vs. 80% of other children, *p *= 0.01). These children were more likely to disagree with either the statement "I am worried that I may become overweight" (*p *= 0.039) or "I am worried that my weight is not good for my health" (*p *= 0.009). When asked whether they were concerned about becoming overweight, 54% of African American children disagreed, compared to only 16% of Latino and Native American children. Similarly, 54% of African-American children disagreed that they were concerned that their weight was unhealthy, compared to only 12% of Latino and Native American children (Table 1). The findings from this study support and validate previous findings regarding African American children's perceptions of their body weight [18,19].

**Table 1:** Childrens' attitudes about own weight by race

	African Americans		Other Race		
	**N**	**%**	**N**	**%**	**Exact *p *value** **p-value**

BMI Group at Baseline					0.222

Normal	3	23.1%	1	4.0%	

Overweight	0	0.0%	2	8.0%	

Obese	10	76.9%	22	88.0%	

Whether Child Thinks she/he is overweight ooverOverweight					0.010

No	8	66.7%	5	19.2%	

Yes	4	33.3%	21	80.8%	

What Child Thinks of Own Weight					0.340

Underweight	2	15.4%	4	16.0%	

About Right	5	38.5%	4	16.0%	

Overweight	6	46.2%	17	68.0%	

Child Is Worried About Becoming overweight					0.039

Disagree	7	53.8%	4	16.0%	

Neutral	2	15.4%	4	16.0%	

Agree	4	30.8%	17	68.0%	

Own Weight is Unhealthy					0.009

Disagree	7	53.8%	3	12.0%	

Neutral	0	0.0%	6	24.0%	

Agree	6	46.2%	16	64.9%	

### Parent's report of child's weight

Twenty-nine parents between ages 31 and 47 years old, with a mean age of 37.5 years (± 4.3) completed the survey. Four parents who had multiple children enrolled in the program completed a survey for each child. All but two parents were the same race as their child, so that nearly one-quarter (24%) of the parents were African-American, two-thirds (69%) were Latino, and the remaining two parents were Native-American and White.

The parents of nearly two-thirds (65%) of the children reported that the child was overweight, while the rest reported their child was underweight or the right weight (Table 2). This did not change when the children whose actual BMI was normal or underweight were excluded. When those children were excluded so that all of the children had a BMI score that indicated that they were overweight or obese, the parents of 18% of the children report that the child was underweight and 18% report that the child was normal weight.

**Table 2:** Parent report of child's weight

	ALL PARENTS		AFRICAN-AMERICAN		OTHER RACE		
	**N**	**%**	**N**	**%**	**N**	**%**	**Exact *p*-value**

Parent Feels Child is:							0.653

Underweight	4	10.8	2	18.2	2	7.7	

About Right	9	24.3	3	27.3	6	23.1	

Overweight	24	64.9	6	54.5	18	69.2	

Parent is Worried that Child 's weight is unhealthy							0.399

Disagree	11	29.7	5	45.5	6	23.1	

Neutral	3	8.1	0	0.0	3	11.5	

Agree	23	62.2	6	54.5	17	65.4	

Parent is Worried that Child Will Become Overweight							0.006

Disagree	13	35.1	8	72.7	5	19.2	

Neutral	0	0.0	0	0.0	0	0.0	

Agree	24	64.9	3	27.3	21	80.8	

African-American parents were less likely to report that they were concerned that their child would become overweight compared to other parents (27% vs. 81%, *p *= 0.006). African-American parents were also less likely to report that their child's weight was unhealthy compared to other parents (46% vs. 77%, *p *= 0.069).

**Figure 1:**
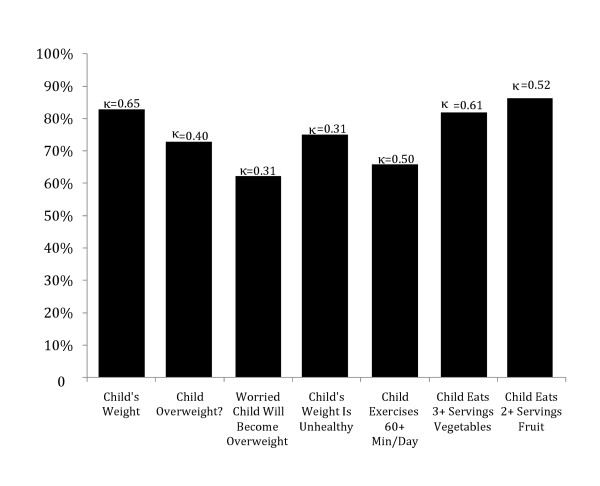
**Percent of parent-child dyads in which responses about child's behaviors agree**.

Figure 1 shows the level of agreement for each of the questions to which both parents and children responded, as well as the Cohen's kappa value (κ) for the level of agreement [17]. The values for Cohen's kappa range from 0 (no agreement) to 1 (perfect agreement) and are adjusted for the distribution of the responses. A value of 0.7 or higher indicates good agreement and a value of 0.8 indicates excellent agreement. None of the measures reaches the threshold for good agreement.

Both parents and children were asked what they thought of the child's weight (underweight, about right, or overweight). Parents and children agreed in 81% of the dyads (κ = 0.65).

The results show that parent reports of their children's behaviors differ in 16%-38% of the dyads. Both parents and children were asked what they thought of the child's weight (underweight, about right, or overweight). Parents and children agreed in 81% of the dyads (κ = 0.65). More than one-quarter (27%) of the parent-child dyads disagreed about whether the child's weight was unhealthy. Parents and children showed the highest disagreement (38%) about whether they were worried that the child would *become *overweight.

When reporting on the child's health behaviors, reporting was more similar on the diet-related items than on exercise. More than 80% of parent-child dyads agreed about whether the child eats three servings of vegetables per day (81%) or eats two servings of fruits per day (84%). In contrast, only 65% of dyads agreed on whether the child exercised at least 60 min per day (Figure 1).

## Discussion

The findings from this study support and validate previous findings about weight related perceptions among overweight children [13,14,18,19]. We found that African-American children were less likely to report that they were overweight than other children. African American children also reported being less concerned about their weight posing a risk to their health compared to other children.

Boyington and colleagues explored the cultural attitudes and perceptions toward body image, food, and physical activity among 12 overweight African American girls, aged 12-18 years. The girls reported that "a healthy body size was one with which an individual felt comfortable" [18]. Subjects reported that weight and body size was determined by the individual and influenced by the opinions of family and peers. Each girl's reference for body size was their family and cultural group which suggest that weight and body size are influenced by culturally based perceptions [18]. This qualitative study found that these girls tolerated heavier body weight and perceived less social pressure to lost weight, resulting in infrequent pursuit of healthy lifestyle changes.

We found that African-American parents were more likely to report their overweight child's weight as underweight or normal and less likely to report they felt their child's weight was unhealthy. This is consistent with perceptions reported in other studies, where a common finding is that parents of overweight children often perceive their child as being at an appropriate weight [13,14,19].

Burnet and colleagues assessed weight related beliefs in urban African American youth and their parents prior to developing a family based intervention to prevent childhood obesity and type 2 diabetes. They found that African American parents defined overweight in functional terms than by measurement or charts. The parents reported that "bigger people are built differently; charts do not always apply". The parents also reported that "larger-framed people would appear unhealthy if they conformed to standardized body mass index charts" [19]. Children relied on physical appearance to define overweight, describing a healthy weight as "medium sized, not too skinny, not too thick" [19].

These findings are concerning because excess weight in childhood is associated with increased risk of adult obesity and diabetes. Parental perceptions of a child's weight may influence a parent's efforts to encourage lifestyle changes for their child [3]. Lifestyle behaviors of African American parents and children may be responses to historical, social, and cultural influences that affect their personal health beliefs, attitudes and perceptions [20]. Identification of health beliefs must be targeted in order to provide a supportive environment to promote healthy behaviors in families.

Limitations to this study include the use of a questionnaire that was not tested for reliability and validity. Some self-reported data may be inaccurate because the subjects may have reported what reflected positively on their own abilities, knowledge, and health beliefs. Children younger than 10 years of age may have experienced difficulty interpreting the questions.

Though the small sample size limits the study's generalizability, the perceptions reported point to key areas for further study. In this sample of children and parents, the findings imply that perception of weight and healthy lifestyle may be influenced by cultural beliefs and personal influences.

## Conclusions

This study's findings indicate that future intervention efforts should assess children's and parents' perception of overweight status and diabetes risk and these factors should be considered when developing prevention interventions for families with youth at risk for diabetes. Identification of beliefs, knowledge, and skills must be targeted, as well as strengths augmented in the community in order to provide a supportive environment to promote healthy nutrition and physical activity behaviors in families.

## Competing interests

The authors declare that they have no competing interests.

## Authors' contributions

EV conceived of the study, participated in its design and coordination and drafted the manuscript. TB participated in the design of the study and performed the statistical analysis. AC participated in the design of the study. All authors read and approved the final manuscript.
